# Tuberculin skin test and ELISPOT/T. SPOT.TB in children and adolescents with juvenile idiopathic arthritis

**DOI:** 10.1186/1546-0096-12-17

**Published:** 2014-05-22

**Authors:** Flavio Sztajnbok, Neio L F Boechat, Samantha B Ribeiro, Sheila K F Oliveira, Denise C N Sztajnbok, Clemax C Sant’Anna

**Affiliations:** 1 Pediatric Rheumatology Division, Universidade Federal do Rio de Janeiro, Rua Bruno Lobo 50, Cidade Universitária, 21941-912 Rio de Janeiro, Brasil; 2 Pneumology Division, Universidade Federal do Rio de Janeiro, Rua Rodolpho Paulo Rocco 255, Cidade Universitária, 21941-913 Rio de Janeiro, Brasil; 3 Molecular Biology Division, Multidisciplinary Laboratory, Hospital Universitário Clementino Fraga Filho, Universidade Federal do Rio de Janeiro, Rua Rodolpho Paulo Rocco 255, Cidade Universitária, 21941-913 Rio de Janeiro, Brasil; 4 Pediatric Infectious Diseases Division, Universidade do Estado do Rio de Janeiro, Avenida 28 de setembro 87, Vila Isabel, 20551-030 Rio de Janeiro, Brasil; 5 Pediatric Pneumology Division, Universidade Federal do Rio de Janeiro, Rua Bruno Lobo 50, Cidade Universitária, 21941-912 Rio de Janeiro, Brasil

## Abstract

**Background:**

There are controversies regarding the accuracy of the tuberculin skin test (TST) and methods based on the production of interferon gamma by sensitized T cells for the diagnosis of latent tuberculosis infection (LTBI) in pediatrics and immunosuppressed patients. Our objectives are to study TST and ELISPOT/T. SPOT.TB in the diagnosis of LTBI in children and adolescents with JIA undergoing methotrexate, the correlation between both and the sensitivity and specificity of T. SPOT.TB.

**Methods:**

This is an observational prospective longitudinal study in which children and adolescents with JIA undergoing methotrexate therapy were assessed for clinical and epidemiological data for LTBI, in addition to performing TST and T. SPOT.TB at baseline and after 3 and 12months.

**Results:**

There were 24 patients. The prevalence of LTBI at inclusion was 20.8%, the incidence after initiation of immunosuppressions 26.3% and the prevalence at the end of the study 41.6%. Epidemiological history positive for TB showed a relative risk of 2.0 for the development of LTBI. Only 2 patients had positive T. SPOT.TB but only in one it was useful for detecting early LTBI. T. SPOT.TB presented a sensitivity of 10%, specificity of 92.8%, and low correlation with TST. No patient developed TB disease at a mean follow-up of 47months.

**Conclusions:**

We found a high prevalence of ILTB that doubled with immunosuppression and that epidemiological history was an important relative risk. T. SPOT.TB showed low sensitivity and high specificity, and no superiority over TST. There was low agreement and little influence of immunosuppression on the results of both tests.

## Background

It is estimated that one third of the world’s population is infected with *Mycobacterium tuberculosis* (*M. tb*) and 15-20% of new cases occur in children and adolescents [1]. In most cases, the infection remains latent. Nevertheless, latent tuberculosis infection (LTBI) can persist with risk of progression to disease in about 10% in the general population and 15-43% in the pediatric age group and is an important cause of morbidity and mortality, especially in immunossupressed patients [2,3]. LTBI is the clinical syndrome caused by exposure to *M. tb* followed by infection, evidenced by the presence of positive tuberculin skin test (TST) in the absence of clinical and radiological signs and symptoms of disease activity [4,5]. To confirm the diagnosis of LTBI, epidemiological data, personal and familiar history, physical examination and laboratorial results should be considered. There is no confirmatory test for the diagnosis of LTBI and TST is still considered the gold standard, despite some limitations. In immunosuppressed patients, TST may present lower sensitivity and false negative results may occur, making LTBI diagnosis even more difficult [6].

Diagnostic tests *in vitro* using whole blood to assess the production of interferon-gamma (IFN -γ) by previously sensitized lymphocytes were developed in order to assist the diagnosis of tuberculosis (TB). These tests, known as IGRA (interferon-gamma release assay), are commercially available and the most frequently used are the QuantiFERON-TB In-Tube® (enzyme linked immunosorbent assay) and T. SPOT.TB ®/ELISPOT (enzyme linked immunospot assay). They require just one visit and are not performed directly on the patient, reducing the possibility of occurring adverse effects, booster effect when repeated and abscence of the patient at the time of reading. However, they are expensive and require specialized laboratories.

In general, different studies have shown 60-80% agreement comparing TST and IGRAs in adults [7]. Some authors [8,9] suggested that T. SPOT.TB presents better sensitivity and specificity than TST in children. But few studies have been conducted in immunosuppressed patients. The T. SPOT.TB assay has shown superiority compared to TST sensitivity and specificity for diagnosis of active TB in immunosuppressed patients [10]. However, most studies have been conducted in countries with low TB incidence and in adult population [7,8]. Studies to determine the sensitivity and specificity of IGRAs in immunocompromised children, especially from endemic areas, are still lacking.

Juvenile idiopathic arthritis (JIA) is the most common cause of chronic arthritis in pediatric age and its pathogenesis is associated with changes in the immune system [11]. This may explain the great predisposition of JIA patients to infections, beyond the fact that they frequently use immunosuppressive drugs as treatment [12]. In adults, rheumatoid arthritis itself, regardless the type of treatment, may be associated with increased incidence of TB, and it raises with the use of immunosuppressive drugs, especially the modern biologic agents, but no data is known for children [13].

BCG is the only available vaccine for TB and provides protection against meningitis and disseminated forms. The World Health Organization (WHO) recommends that children from high-burden countries should be vaccinated at birth, a practice common in most of these countries. In low-burden countries this practice is restricted to children from high-risk groups for TB [4].

Early diagnosis and treatment of LTBI are challenges to be achieved in order to control TB worldwide. There is a need for more studies on the accuracy of diagnostic methods for TB in children and immunosuppressed patients, especially in high-burden countries. This study aimed to evaluate the frequency of LTBI and the sensitivity and specificity of the T. SPOT.TB compared to TST in the diagnosis of LTBI in patients with JIA before and after the use of methotrexate (MTX), as it is the first-choice second-line agent most frequently used in JIA [14], in a high-burden country where most children receive BCG at birth.

## Methods

This was an observational prospective longitudinal one-year duration study approved by the local IRB. Children and adolescents with JIA (Edmonton Revision, 2001 [15]) and indication to use MTX without previous or current diagnosis of TB disease and no prior use of immunosuppressive drugs in the previous six months, were consecutively included from March 2008 to September 2011. All patients received MTX at a dosage of 10–15 mg/m^2^/week as the first second line drug after nonsteroidal antiinflammatory drug (NSAID) failure, as the usual practice in our unit. If during the one-year follow-up corticosteroid or a biologic agent was necessary, the patient could continue in the study. However patients with indication to start a biologic agent were not included as these drugs are known to increase TB incidence [13] and they were already using MTX.

All patients answered a questionnaire to assess epidemiological data regarding TB at the first visit (T0). The intention was to evaluate contact with suspected cases of TB, presence of signs and symptoms suggestive of TB in patients and their families and previous treatment of LTBI or active disease. The questionnaire included previous BCG vaccination and the presence of the charactheristic skin scar (later confirmed by a physician), results of previous TST eventually performed, previous history, treatment or signs and symptoms of TB (patient and the family: unexplainned prolonged fever, prolonged cough, pulmonary or pleural disorders), socio-economic conditions of the residence and neighborhood and contact with persons with TB.

At T0 chest radiography, TST, and basic laboratory tests such as complete blood count, acute phase reactants, assessment of renal and hepatic function, urinalysis, HIV and the T. SPOT.TB test were ordered. TST and T. SPOT.TB were also performed in the 3^rd^ (T3) and 12^th^ (T12) months. Chest radiography was repeated if either of the TB related tests became positive.

TST consisted of intradermal injection of 0.1 ml of purified protein derivative (PPD RT 23 2 IU) of *M. tb*, according to local practice. Patients were considered to have a positive TST if an induration ≥ 5 mm diameter was present 48–72 hours after inoculation [4]. The T. SPOT.TB/ELISPOT test was performed according to the manufacturer’s recommendations. The test included sections with negative control (C-: no mitogens or antigens), sections with positive control (C +: phytohemagglutinin) and sections to test two antigens (ESAT-6 and CFP-10). Spot-forming units (SFUs) were counted with the aid of a magnifying glass. Interpretation of the results was made as follows: when the negative control contained ≤ 5 SFUs, this value was subtracted from the number of SFUs found in the section containing the antigens ESAT-6 and CFP-10, and so: positive test ≥ 6 SFUs for either antigen; negative test ≤ 6 SFUs for both antigens and ≥ 20 SFUs in the positive control section; indeterminate test ≤ 6 SFUs for both antigens and ≤ 20 SFUs in the positive control section. In case the negative control section presented with ≥ 6 SFUs, the test was considered positive if the number of SFUs in either antigen was greater than twice the number found in the negative control section. Samples of children with bacteriological TB were used as external control. The techinican performing the tests had no knowledge of clinical or laboratory data of the patients.

Descriptive statistics were used to obtain absolute frequencies and percentages for qualitative data, as well as means, medians, and standard deviations for quantitative data. The relationship between epidemiological history and the presence of LTBI was evaluated by chi-square test (significant if p value was less than 0.05). The sensitivity, specificity, positive predictive value (PPV) and negative predictive value (NPV) were calculated from 2×2 contingency tables, using the free program “Openepi”. The *kappa* statistic was adopted to quantify the agreement between the results of TST and T. SPOT.TB using “Statistical Package for Social Sciences” (SPSS) version 16.0. The agreement was considered poor for *kappa* values less than 0.41, moderate between 0.41 and 0.60, substantial between 0.61 and 0.80, and excellent when *kappa* was more than 0.81.

## Results

The study included 29 patients with JIA but because of non-adherence to the protocol before visit T3, five patients were discontinued and, thus, 24 patients remained in the study. Epidemiological and clinical data of the patients at baseline are shown in Table 1. There were 17 female patients (70.8%). Ten patients (41.7%) were polyarticular rheumatoid-factor negative, nine (37.5%) oligoarticular persistent and five (20.8%) systemic. The mean time for MTX onset was 30 months after diagnosis and 35 months after disease onset. All children received BCG vaccine at birth as a current practice in Brazil. Twenty patients were using NSAIDs and four were out of medications at the time of MTX onset. During the one-year follow-up, no patient needed to use corticosteroid or a biologic agent.

**Table 1:** Epidemiological and clinical data from patients with JIA (n = 24)

**Variables**		**n**	**%**
**Gender**	Female	17	70,8
	Male	7	29,2
**JIA subtypes**	Polyarticular rheumatoid factor negative	10	41,7
	Oligoarticular persistent	9	37,5
	Systemic	5	20,8
**Patients’ age at JIA diagnosis (range; mean; median)**	20-216		
	(100±50; 92)		
**Timeframe between JIA diagnosis and inclusion in the study (range; mean; median)**	1-108		
	(30±38; 9)		
**Disease duration: timeframe between JIA onset and inclusion in the study (range; mean; median)**	2-108		
	(35±37; 23)		
**Patients’ age at inclusion (range; mean; median)**	36-217		
	(130±51; 119)		
**Epidemiological history of contact with TB at inclusion (T0)**	yes	8	33,3
	no	16	66,6

Time is referred in months.

At T0, eight patients had positive epidemiological history for TB and five of them developed LTBI. Of 16 patients without a positive epidemiological history, five developed LTBI. The relative risk was 2 for patients with epidemiological history for TB compared to those without. None of the patients with positive results for T. SPOT.TB had a positive epidemiological history for TB. All 24 patients in the study had normal chest radiography at T0 and there were no reports of new positive epidemiological history of TB at T3 and T12.

At T0, before MTX onset, five patients already had a positive TST. At T12, no patient had developed TB disease and 10 had developed LTBI. Thus, LTBI was diagnosed in 5/24 (20.8%) patients at T0 and 5/19 (26.3%) patients after immunosuppression onset, what means 10/24 (41.6%) patients from T0 to T12. All patients with positive TST were treated for LTBI with isoniazid for six months, and there was no occurrence of toxicity in any of them. All those with conversion of TST had a new chest X-ray, which was normal in all cases. No patient had a TST in the previous 2years before T0.

The agreement between TST and T. SPOT.TB could not be assessed at T0 due to the fact that all results were negative for the T. SPOT.TB. At T3, two patients had positive T. SPOT.TB tests and, at T12, one of them had a negative test. Thus, there was a poor correlation between the two tools (κ = 0.11 and 0.16, respectively).

To evaluate the sensitivity and specificity of the T. SPOT.TB, we considered TST value ≥ 5 mm as the gold standard. At T3 the sensitivity of T. SPOT.TB was 20% (CI 95% = 3.6 to 62.4) and the specificity 89.4% (CI 95% = 68.6 to 97), and at T12 12.5% (CI 95% = 2.2 to 47) and 100% (CI 95% = 80.6 to 100), respectively. Taking into account that 10 patients had LTBI, the final sensitivity of the T. SPOT.TB was 10% (CI 95% = 1.7 to 40.4) and specificity was 92.8% (CI 95% = 68.5 to 98.7). In this scenario, the PPV of the T. SPOT.TB was 50% (CI 95% = 9.4 to 90.5) and the NPV was 59% (CI 95% = 38.7 to 76.7). A ROC curve for T. SPOT.TB showed an area under the curve of 0.51 (p value 0.81).

No other immunosuppressive therapy was initiated and no MTX discontinued during the one-year study follow-up. In the last appointment in March 2013, after a mean follow-up of 3years and 11months, no patient in the study had developed active TB. Table 2 shows clinical and epidemiological data besides TST and T. SPOT.TB results for each subject in the study.

**Table 2:** Epidemiological and clinical data besides TST and T. SPOT.TB/ELISPOT results from the 24 cases

**Case/gender**	**JIA**	**Age at diagnosis (months)**	**Time Dx-T0 (months)**	**Age T0 (months)**	**T0**	**T3**	**T12**	**Follow up (months)**	**LTBI**
1	P	72	16	88	TST 0	TST0	TST 0	57	
M					Elispot -	Elispot -	Elispot -		
2*	O	20	90	110	TST 0	TST 4	TST 10	58	yes
F					Elispot -	Elispot -	Elispot -		
3	P	152	2	154	TST 7	TST 0	TST 0	59	yes
F					Elispot -	Elispot -	Elispot -		
4	O	192	24	216	TST 0	TST 0	TST 0	59	
F					Elispot -	Elispot -	Elispot -		
5*	P	130	6	136	TST 5	TST 19	TST 12	55	yes
F					Elispot -	Elispot -	Elispot -		
6*	O	88	1	89	TST 0	TST 0	TST 0	56	
M					Elispot -	Elispot -	Elispot -		
7*	S	102	2	104	TST 0	TST 10	TST 10	56	Yes
F					Elispot -	Elispot -	Elispot -		
8	O	84	106	190	TST 14	TST 10	TST 15	56	Yes
F					Elispot -	Elispot -	Elispot -		
9	O	108	99	207	TST 13	TST 15	TST 13	56	Yes
M					Elispot -	Elispot -	Elispot -		
10	O	86	30	116	TST 0	TST 0	TST 0	55	
F					Elispot -	Elispot -	Elispot -		
11	S	113	1	114	TST 0	TST 0	TST 0	53	
F					Elispot -	Elispot -	Elispot -		
12	S	24	46	70	TST 0	TST 0	TST 0	37	
F					Elispot -	Elispot +	Elispot -		
13	P	139	4	143	TST 0	TST 0	TST 10	52	Yes
M					Elispot -	Elispot +	Elispot +		
14*	P	111	12	123	TST 3	TST 3	TST 0	51	
M					Elispot -	Elispot -	Elispot -		
15*	O	85	108	193	TST 0	TST 0	TST 0	50	
F					Elispot -	Elispot -	Elispot -		
16	S	54	48	102	TST 1	TST 0	TST 0	29	
F					Elispot -	Elispot -	Elispot -		
17	O	60	90	150	TST 0	TST 0	TST 0	29	
F					Elispot -	Elispot -	Elispot -		
18	S	66	1	67	TST 0	TST 0	TST 0	28	
F					Elispot -	Elispot -	Elispot -		
19*	P	156	32	188	TST 0	TST 0	TST 23	25	Yes
M					Elispot -	Elispot -	Elispot -		
20	P	160	1	161	TST 0	TST 0	TST 0	19	
F					Elispot -	Elispot -	Elispot -		
21	P	61	1	62	TST 0	TST 0	TST 0	18	
F					Elispot -	Elispot -	Elispot -		
22	O	35	1	36	TST 0	TST 10	TST 10	60	Yes
F					Elispot -	Elispot -	Elispot -		
23	P	96	2	98	TST 0	TST 0	TST 0	57	
F					Elispot -	Elispot -	Elispot -		
24*	P	216	1	217	TST 5	TST 4	TST 2	57	Yes
M					Elispot -	Elispot -	Elispot -		

Legend:

• JIA = JIA subtypes: O = oligoarticular JIA; P= polyarticular JIA; S= systemic JIA.

• (*) = epidemiological hystory positive for TB.

• TST: in millimeters.

• T.SPOT.TB/ELISPOT : (-) = negative; (+) = positive.

• T0: study onset; T3: 3rd month; T12: 12th month.

## Discussion

To our knowledge, this study was the first to assess the performance of TST and T. SPOT.TB in pediatric patients with a rheumatic disease in a high-burden country. Brazil presents a high incidence of TB, around 38 cases per 100.000 inhabitants in 2011 [16].

In our study, the epidemiological history for TB doubled the relative risk for the development of LTBI compared to patients with negative epidemiological history. In a systematic review in which 40% of the studies were conducted in countries where TB was endemic, it was found an association between positive epidemiological history and responses in TST and IGRAs [17]. The authors suggested that an important marker to be considered for the diagnosis of LTBI would be the degree of exposure of the patient, i.e. location, number of hours of exposure to a person with TB and bacillary load. Unfortunately, in our study, such an assessment was virtually impossible because the lack of information provided by the assisted population. Thus, our study considered only yes/no responses concerning exposure.

The conversion of TST usually occurs about two to 12 weeks after infection and the T. SPOT.TB between four and 22 weeks [18]. In one patient (#13), T. SPOT.TB presented a positive result prior to TST conversion and, at least in this case, T. SPOT.TB was sensitive enough to identify LTBI in advance. In the other patient (#12), T. SPOT.TB became negative at T12 and TST remained negative. One explanation for this latter patient could be the occurrence of a booster effect because IGRA in T3 was performed sometime after TST in this patient. This is a rare but possible event [19,20].

No patient developed active TB during the one-year follow-up of the study. All patients had a prolonged follow-up after this initial proposed study time (one year), with a mean time of 3 years and 11 months, and although in this phase there were patients who started biologic agents, none of them developed active TB. We believe that this fact was possibly due to the early treatment of LTBI [21].

We did not find national or international data to compare our high rates of LTBI as most of the data available refers to active TB. The most comparable to ours, as conditions of patients at risk for acquiring LTBI, were reported rates of LTBI in 61.5% of subjects in a prison hospital, 40% of shelter residents, and 20% of injection drug users [22]. Besides the high incidence of TB in Brazil, additional factors that could explain the high rates of LTBI in our study are the immunosuppression associated with JIA itself, patients’ frequent exposure to health services and possible TB, and the use of MTX.

Our study showed poor correlation between the results of TST and T. SPOT.TB. The few available studies showed that such an agreement appears to be low in countries where TB is endemic, unlikely in countries with low incidence, and this could be explained by the high sensitivity inherent to TST revealing false negative IGRAs [5,19,23,24]. It should be noted that patients received BCG at birth, many years before study participation, so BCG very probably did not influence TST results. It is known that TST positivity associated to BCG in the neonatal age decreases rapidly in a few years [25]. TST positivity could not be explained by a booster effect, as no patient had TST performed in the two years prior to the study onset.

Immunosuppression may affect the sensitivity of both TST and IGRAs [23,26]. Although in immunosuppressed adults IGRAs showed higher sensitivity than TST [21,27,28], some authors found lower values for both TST and IGRAs in patients with rheumatoid arthritis using immunosuppressants compared to those not using immunosuppressants [23]. For the TST cut-off value of 5 mm as used in Brazil [4], IGRAs showed high specificity and low sensitivity. At this cut-off, TST performed better than T. SPOT.TB. In our study, it was difficult to evaluate the role of immunosuppression on the results of T. SPOT.TB, since all were negative at T0, before MTX onset. Between T3 and T12 sensitivity, which was already low, slightly decreased. It was not possible to explain why four patients showed conversion of TST during the study and the T. SPOT.TB remained negative. Although TST and T. SPOT.TB evaluate different responses, it would be expected that immunosuppression could influence TST more often than IGRAs, since the former depends on the individual hypersensitive and T-cell proliferation [25,29]. Therefore, in our patients, immunosuppression from MTX did not influence the results of T. SPOT.TB.

T. SPOT.TB in children from endemic areas has shown lower sensitivity and higher specificity than TST [30,31]. It had been shown that IGRAs presented better accuracy than the TST for the diagnosis of TB in immunocompromised children [17], but a recent study showed that IGRAs in children and immunosuppressed patients might present false-negative results in endemic areas for TB [32]. Throughout our study, T. SPOT.TB showed low sensitivity but high specificity. If a positive T. SPOT.TB was used for the diagnosis and indication for treatment of LTBI in our patients, only two of them would have been diagnosed as LTBI and treated. This could mean that, in a high-burden setting, patients might be untreated. On the other hand, it has been proposed that in low-burden settings replacing TST with IGRA for determining LTBI could allow a reduction in the number of patients receiving treatment [28].

A study with epidemiological characteristics similar to ours compared the use of TST and T. SPOT.TB in the diagnosis of LTBI in adult patients with rheumatoid arthritis and indication for use of biological agents. The specificity of the T. SPOT.TB ranged from 87% to 90% and NPV from 94.4% to 100%, with similar sensitivity to TST. Authors suggested that the low sensitivity of T. SPOT.TB in endemic areas for TB could be explained by the fact that the antigens in the T. SPOT.TB plate, although specific for *M. tb*, represented a small portion of numerous antigens, and more reliable results could be achieved by adding new antigens to the method [33]. In Gambia, a TB- endemic region, T. SPOT.TB and TST had similar results in the diagnosis of LTBI in children vaccinated with BCG at an early age. TST and IGRA in conjunction increased sensitivity in only 10% [20].

Our study had limitations, but many of them bring us to real-life conditions, especially in developing countries. The low prevalence of JIA might explain the difficulty to include patients [11] and, unfortunatelly, there was no possibility to test the real state of immunosuppression in patients by assessing CD4 and CD8 counts.

Recently, WHO recommended that IGRAs should not be used in low- and middle-income countries (generally those with higher TB burden), as there are insufficient data and evidence on the performance of IGRAs in these populations [4]. IGRAs have similar performance to TST but are more expensive and complex to perform [4,34] and, therefore, TST should be preferred in these settings. However, data from pediatric population and immunosuppressed individuals who could benefit from IGRAs are limited and more studies with large sample sizes are desirable.

## Conclusion

In conclusion, we found a high frequency of LTBI among patients with JIA, which doubled after one year of MTX therapy. There was a low agreement between TST and T. SPOT.TB, low sensitivity and high specificity for the T. SPOT.TB, and small influence of immunosuppression related to MTX use on the performance of both. Even with MTX, no cases treated for LTBI developed active TB. There was no superiority of T. SPOT.TB compared to TST for the diagnosis and monitoring of LTBI in childen with JIA using MTX.

## Competing interest

The authors declare that they have no competing interest.

## Authors’ contribution

FS and all co-authors participated in the conception, design, analysis and interpretation of data. SBR also performed all Elispot tests. All authors read and approved the final manuscript.
